# Simulation-based evaluation of two insect trapping grids for delimitation surveys

**DOI:** 10.1038/s41598-022-14958-5

**Published:** 2022-06-30

**Authors:** Hui Fang, Barney P. Caton, Nicholas C. Manoukis, Godshen R. Pallipparambil

**Affiliations:** 1 NSF Center for Integrated Pest Management, North Carolina State University, 1730 Varsity Drive, Suite 110, Raleigh, NC 27606 USA; 2 Plant Protection and Quarantine, Animal and Plant Health Inspection Service, United States Department of Agriculture, 920 Main Campus Drive, Suite 400, Raleigh, NC 27606 USA; 3 Daniel K. Inouye U.S. Pacific Basin Agricultural Research Center, Agricultural Research Service, United States Department of Agriculture, 64 Nowelo Street, Hilo, HI 96720 USA

**Keywords:** Invasive species, Ecological modelling

## Abstract

In the United States of America, delimitation trapping surveys with square grids have been used for decades for exotic insects without rigorous evaluation. We used simulations to investigate the effectiveness of two representative designs: an 8-km grid for *Acrolepiopsis assectella* (leek moth) and a 14.5-km grid for *Ceratitis capitata* (Mediterranean fruit fly, “Medfly”). We investigated grid compositions and design factors, measuring performance as the mean probability of pest capture over all traps, *p*(capture), and designed improved grids for both species. For the standard designs, *p*(capture) was 0.86 for leek moth and 0.71 for Medfly, with the latter performing better due to greater lure and trap attractiveness. For both designs, 86 percent or more of mean *p*(capture) came from core area captures. Egress testing indicated that both grids were oversized. An improved grid for leek moths would use 177 traps in a 4.8-km diameter circle, which had mean *p*(capture) = 0.73 and reduced the cost by 80 percent. The best Medfly grid was a 4.8-km diameter circle with 232 traps, which gave mean *p*(capture) of 0.66 and reduced the cost by 86 percent. Simulation may be used to improve trapping survey plans, often saving significantly on costs while maintaining survey performance.

## Introduction

When adventive populations of exotic pests are detected delimiting surveys are usually an important first step in the response, to determine the boundaries of the infested area^1,2^. The two purposes of delimiting surveys are to confirm the presence of the pest population and to determine the size of the area occupied^3^. Containing the adventive population during delimitation is also important^3^ and is usually a priority in setting up quarantine areas^4^. For many mobile insects delimitation involves trapping^5^.

**Figure 1:**
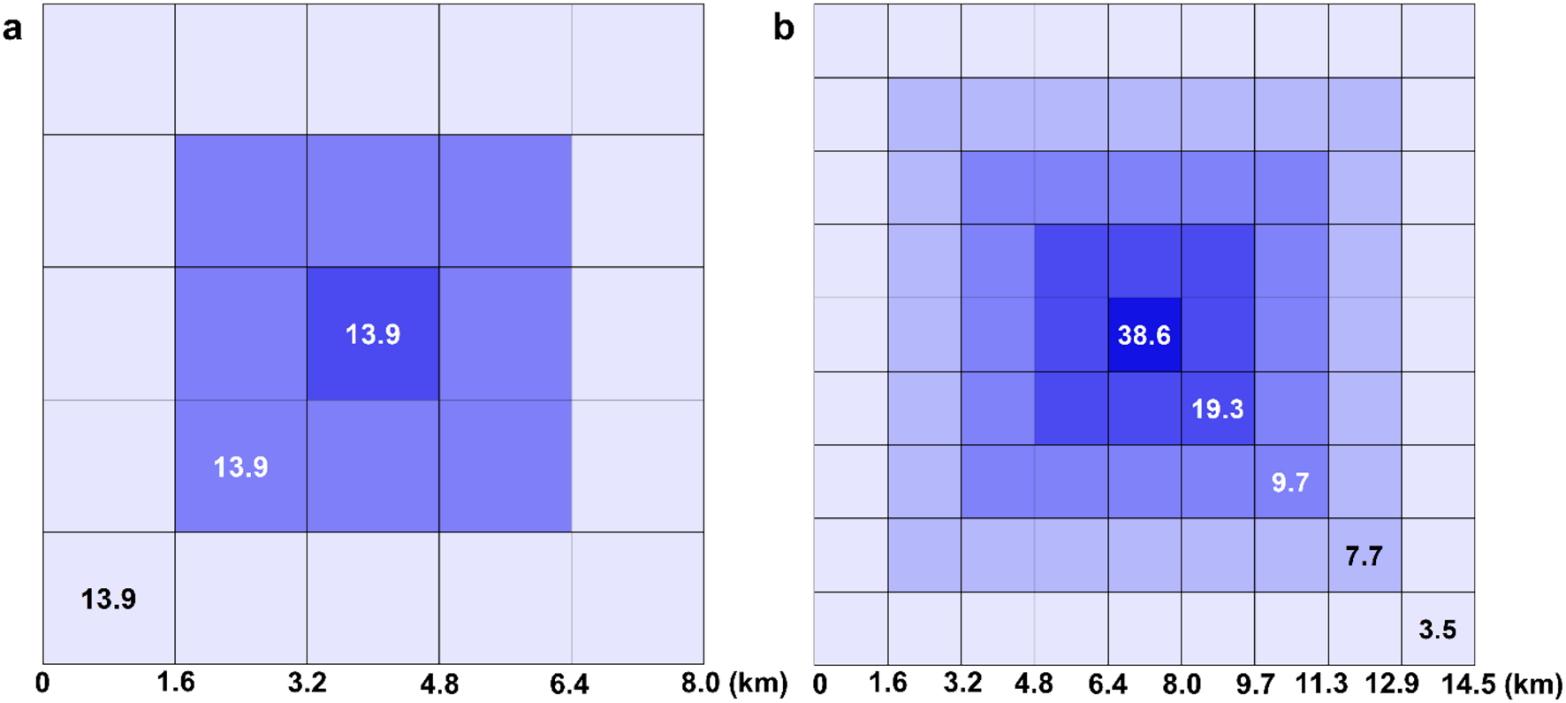
Examples of published delimiting survey trapping grids for: (**a**) The leek moth, with a uniform trap density, and (**b**) the Medfly, with variable trap densities. Each cell represents 2.59 km^2^ (one square mile); the number in each cell shows approximate trap density (no. per km^2^) and each color indicates a band.

In the United States of America, delimiting survey designs are only minimally customized based on the pest of concern or the trapping system. Typically, design choices come down to one of two standard sizes of square-shaped trapping grids. The smaller of the two has side lengths of 8.0 km (5 mi; hereafter, “8-km grid”) (Fig. 1a) and has been proposed for small moths, such as leek moth (*Acrolepiopsis assectella* (Zeller))^6^, plum fruit moth (*Grapholita funebrana* (Treitschke))^7^, summer fruit tortrix moth (*Adoxophyes orana* (Fischer von Roslerstamm))^8^, and *Spodoptera* spp.^9^. The other common grid has side lengths of 14.5 km (9 mi; hereafter, “14.5-km grid”) (Fig. 1b) and has been specified for use with nearly every fruit fly of concern^10^, including Mediterranean fruit fly (Medfly, *Ceratitis capitata* (Weidemann)), Oriental fruit fly (OFF, *Bactrocera dorsalis* (Hendel)), Mexican fruit fly (Mexfly, *Anastrepha ludens* (Loew)), and melon fly (*Zeugodacus cucurbitae* (Coquillett)).

The dispersal ability of the target pest influences the chosen grid size^11,12^, but many other factors known to affect survey performance are often not adequately considered. Trap density is important but trap attractiveness is the most important single factor affecting survey performance^13,14^. Attractiveness is well known to vary by type of trap or lure^16–18^. Consequently, the density (or densities) for a particular trap type with specific attractiveness are likely chosen arbitrarily to achieve expected detection probabilities, while considering supply costs and servicing time and labor^19^. Other potentially important factors include trapping duration, grid shape, resource limitations, and/or operational constraints.

These standard delimiting designs have been used for decades in many cases, but their performance has largely been unquestioned and unstudied. We have seen no reports of failures (insect escape), but the only evidence for their effectiveness is anecdotal, in the form of continued usage without adaptations. Moreover, the creation of these designs was not based on rigorous experimentation or theoretical analysis. The 14.5-km grid was devised by an expert group based largely on collective experience and expectations (PPQ, personal communication). The group dealt with the uncertainty around Medfly dispersal potential by increasing the trapping grid size three-fold to act as a reasonable safety buffer, an amount which was similarly proposed elsewhere for Queensland fruit fly (Qfly, *Bactrocera tryoni* (Froggatt))^20^. The Medfly design incorporates variable densities throughout, but the rationale for that has been lost, except perhaps to limit the traps needed for a grid of that size (PPQ, personal communication). The approach to date for employing delimiting surveys could largely be described as “one-size-fits-all”.

The study of delimiting survey performance in field experiments is lengthy, difficult, and expensive. There are many potentially important experimental factors, and complex interactions between insects, traps, and the environment. Therefore, controlled experiments on just a few important factors might take several years and would likely be limited to studying a single pest. In contrast, simulation models can be an efficient and effective means of investigating ecological phenomena. They are particularly well-suited to situations in which the interactions are complex, and when factor importance is not well understood^21^. Different models have been developed and used to estimate the attractiveness of traps and probability of capture of trapping grids^13,15,22–26^, or to optimize the spatial deployment of traps in integrated pest management^27^. The simulation model, TrapGrid, was created to simulate insect trapping networks and quantify capture probabilities^13^.

Here, we used TrapGrid to evaluate the performance of two representative delimiting survey trapping designs for insects in the USA, focusing on localized adventive populations. Our objectives were to: (1) evaluate the overall performance of the two standard grid designs; (2) deconstruct and manipulate the two grids to better understand how design factors affected performance; (3) investigate the appropriateness of the grid sizes in relation to the dispersal ability of the insects; and (4) use the model to design similarly effective, less costly designs for each species.

## Materials and Methods

### Trapping survey grid designs

The two designs chosen for evaluation were the 8-km grid for leek moth^6^ and the 14.5-km grid for Medfly^10^ (Fig. 1a,b). The leek moth grid used a constant trap density, of ~ 13.9 traps per km^2^ (36 per mi^2^), giving it 900 total traps (Fig. 1a). The Medfly grid had variable density: ~ 38.6 traps per km^2^ (Band 1 (hereafter referred to as the “core”), ~ 18.9 in Band 2, ~ 9.7 in Band 3, ~ 7.7 in Band 4, and ~ 3.5 in Band 5 (Fig. 1b)^10^. The total number of traps was 1,700. Trap density affects overall survey effectiveness and directly impacts survey budgets through supply costs and required daily or weekly monitoring. Theoretically, higher densities should increase the probability of capture, *p*(capture), up to the point where trap capture zones overlap or compete with each other (“interference”)^28^. Practically however, densities are often limited by resource requirements.

### Simulation model

All simulations were performed in TrapGrid, which is a landscape-level, spatially explicit model that simulates a population of insects moving in a defined trapping network^13,29^. The model calculates and reports the daily average escape probability for the population, *p*(escape), which is the mean probability of capturing zero insects. The value of *p*(escape) depends on the spatial distribution of the traps, trap attractiveness, and the distance between insect positions and traps. The mean probability of capture, *p*(capture), is 1 − *p*(escape), which represents the cumulative mean daily probability of capturing any individual. The value of *p*(capture), cumulative to that day, was our standard metric for gauging survey performance in simulations, and this value was averaged over 1,000 iterations. TrapGrid currently does not provide information on where in the grid insects were captured, or how many. Thus, *p*(capture) results encompass a variety of outcomes: capturing one insect looks no different than capturing ten insects.

The expected spread of insect pests in the model is a normal distribution centered from the epicenter^30^ with a standard deviation (σ) proportional to the time (*t*) elapsed, as follows^13^:

1 $$ \sigma \, = { 4 } \times \, \left( {Dt} \right)^{{0.{5}}} $$

### Model parameter specifications

TrapGrid has six key parameters: grid side length, trap density, survey duration (*t*), population size (*N*), diffusion coefficient for the insect (*D*), and trap attractiveness (1/λ). Outbreak locations also can be specified.

### Population size

Population size, *N*, was 50 individuals in all simulations. This reflected a first or second generation of insects early in an outbreak^31^, as befits a delimitation exercise. That value was static in each model run, i.e., no natality or mortality^13^. Further, the version of TrapGrid that we used is not sensitive to the number of individuals in the outbreak, as the average capture probability is being calculated^32^. The number of insects may affect the variances of simulation results, but the average will not change.

### Diffusion coefficient

Insect movement is parameterized as the diffusion coefficient, *D* (m^2^ per day), which is the potential daily area explored by the organism^33^. Reported values range from near zero into the 10,000s^11^.

Leek moth is considered to be a weak flyer^34^. Most moths will fly no more than 100 – 200 m from overwintering sites^35^. The value of *D* was around 500 m^2^ per day based on proposed qualitative guidelines^36^.

Medfly is a moderate disperser^37–39^, despite perhaps having a reputation as being highly motile. The *D* value for Medfly was 5,000 m^2^ per day^12,25^.

### Trap attractiveness

This parameter, 1/λ (m), describes the distance at which a responsive target insect has a 65 percent probability of being lured and caught by a single trap^13^. Hence, 1/λ provides a useful metric for comparing the effectiveness of different trap types, and a few values have been experimentally determined^17,40^. The standard Medfly trap uses the synthetic pheromone, trimedlure, which has 1/λ equal to 7 – 14 m^13^. We used an average of 10 m for Medfly here.

Sticky wing traps baited with a pheromone lure have been recommended for leek moth^6^, but we found no direct information on the attractiveness of this configuration. To try to estimate 1/ λ for this insect we first looked at information for pheromone trapping of the spongy moth, *Lymantria dispar* (L.). That trap and lure had a plume length of about 26 m and capture rates up to about 45 percent, but the reported capture probability was only 0.37 for an insect next to the trap (i.e., 0 m distance)^41^. In comparison, Renou et al.^42^ reported capture rates for leek moth up to 95 percent, and Blatt et al.^43^ found that using only one leek moth trap per farm was effective for detection efforts. Because this pheromone seemed to be relatively strong, we qualitatively estimated^36^ that the trap had high attractiveness, with 1/λ = 20 m. However, because that estimate was uncertain, when investigating the optimal design for leek moth we also tested a separate, lower value of 10 m, for comparison. That value is appropriate for a trap with a pheromone having average attractiveness^36^.

### Outbreak locations

**Figure 2:**
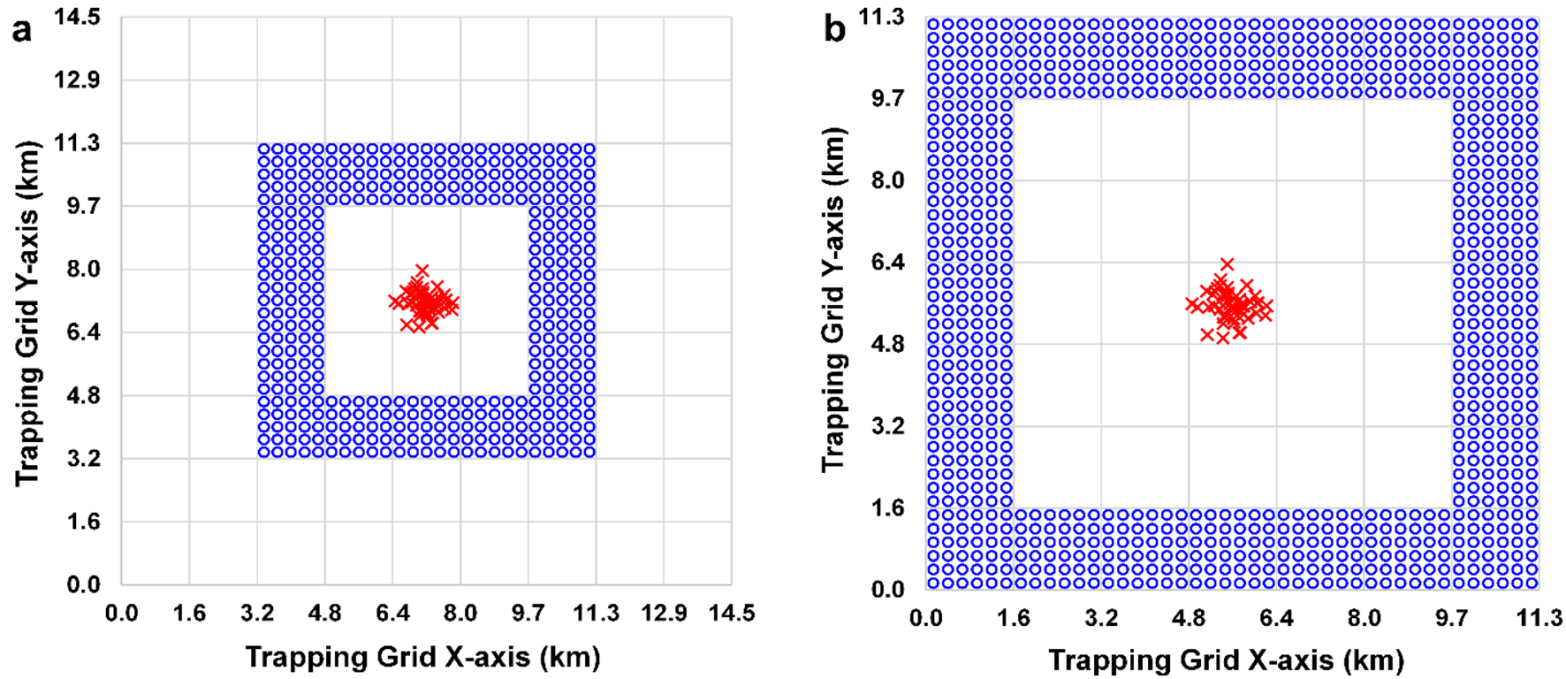
Examples of perimeter designs for band contribution and containment testing. (**a**) Contribution test of Band 3 of the Medfly design with 9.7 traps per km^2^; (**b**) Containment testing grid for the leek moth design with 13.9 traps per km^2^. Each cell is 2.59 km^2^, blue circles represent traps, and red crosses indicate outbreak locations.

In every simulation, we randomized 50 population outbreak locations within the core (2.59 km^2^; see Fig. 2). Doing this helped account for the possibility of source populations not being centrally located in the survey grid.

### Simulation analyses

We used TrapGrid version 2019-12-11^44^. Unless otherwise specified below, simulations were run with the baseline parameters. The survey duration, *t*, in each iteration was 30-d, to reflect a short delimiting activity aimed at providing information for decision making.

#### Comparative grid performance

We evaluated and compared the standard grids of each species, with sizes and densities as stated above. Medfly is considered a more serious pest risk than leek moth and we hypothesized that *p*(capture) should be greatest for the Medfly grid. Also, we expected that grid size differences could be justified by the difference in dispersal abilities of the two species.

#### Evaluating band contributions and density impact

We evaluated grid design factors to better understand how they affected survey performance. This entailed creating grid maps with only single bands for each design (e.g., Fig. 2a)—each of the three bands for leek moth, each of the five bands for Medfly—with trap densities as defined above.

Also, we evaluated full grids with different trap densities. For the leek moth we tested uniform densities of 3.5, 6.2, 9.7, 13.9, 18.9, 24.7, 31.3, and 38.6 traps per km^2^ (these correspond to densities of 9 to 100 traps per square mile). The test for Medfly was more complicated because of its variable density grid. First, we held all outer band densities constant and tested only the core density, starting at 31.3 traps per km^2^ and stopping before *p*(capture) decreased significantly from the value achieved by the standard grid. Then, we repeated this process in successive bands. Finally, we tested a uniform density of 38.6 traps per km^2^ across the entire grid to gauge potential maximum performance.

#### Evaluating population containment

Although not a specific goal in the standard biosecurity definition of delimitation (see above), containment of the population during the survey is important. Containment is usually used as a justification for chosen grid sizes^10,27,37^ and quarantine areas^20,31,45,46^. We think it is also implied that to set an accurate boundary the delimitation survey should be large enough to potentially capture all insects in the population.

The objective here was to determine if the leek moth and Medfly grids were sized appropriately for containing the populations. We simulated each pest with a perimeter trapping grid (1.6 km wide) just larger than the standard grid size (e.g., Fig. 2b). The perimeter for leek moth was set 4.0 km away from the epicenter, and the perimeter for Medfly was 7.24 km away from the epicenter. Default *D* values were used for the two species. The leek moth grid had 13.9 traps per km^2^ in the perimeter, while the Medfly grid used 38.6 traps per km^2^. In these simulations only, we used 1/λ = 30 m (high trap attractiveness) to increase the likelihood of any egressing insects being captured in the simulation; more realistic values for these species might have missed some escapes. We also checked that the results were consistent with the band contribution tests described above.

#### Simulation-based improved grid designs

To derive improved designs based on both survey performance and cost, we tested modifications to trap densities and grid sizes. Every design evaluated was circular, because we previously found that they were more efficient than square ones^47^.

We assessed cost effectiveness using pricing information for traps and servicing. The unit cost for buying and servicing the leek moth traps (sticky wings baited with pheromone) for one month was around $28 (Ontario Ministry of Agriculture, Food & Rural Affairs, personal communication). The same cost for a Medfly trap (ChamP™ or Jackson traps, baited with synthetic pheromone, trimedlure) was $26 (California Department of Food and Agriculture, personal communication). We calculated the return on investment (ROI) as the capture percentage obtained per $1,000 spent.

For leek moth, we tested fourteen alternative designs: seven densities in two grid sizes. The two diameters were 4.8 (i.e., two bands) or 8.0 km (three bands). The densities were 6.2, 9.7, 13.9, 18.9, 24.7, 31.3, and 38.6 traps per km^2^. As mentioned above, we ran these tests with the default value for 1/λ of 20 m, and again with a value of 10 m, to account for uncertainty.

For Medfly, the circular designs used had bands with varying densities. We tested the standard design (but circular) against four alternatives as follows:

1. A 4.8 km diameter grid with the default densities for each included band
2. A 4.8 km diameter grid with the Band 2 density lowered to 13.9 traps per km^2^
3. A 4.8 km diameter grid with the Band 2 density lowered to 9.7 traps per km^2^
4. A 6.4 km diameter grid with the default core density but a reduced, constant density of 6.2 traps per km^2^ in the outside bands

## Results

### Comparative grid performance

For the standard trapping grid designs, *p*(capture) was 0.86 for leek moth, versus 0.71 for Medfly. Medfly grid performance was lower than that of the leek moth grid despite Medfly having greater *D*, a larger grid, and higher trap density in the core. Notably, though, trap attractiveness was greater for leek moth.

### Captures by band and impact of density on performance

#### Band contributions

Overall *p*(capture) values for full grids were less than the sum for all bands (e.g., 0.71 vs. 0.78 for Medfly), because some captures occurred in multiple bands when simulating the full grid (Table 1). The core of the Medfly grid always contributed the most to overall *p*(capture), with a value of 0.67 (94 percent of the total *p*(capture) in the whole grid) on its own (Table 1). When solitary, Band 2 had *p*(capture) = 0.09, and the three other bands combined had *p*(capture) = 0.0 (Table 1).

**Table 1:** The probability of capture for the standard Medfly grid, for different bands, and for the grid with a uniform density of 38.6 trap per km^2^.

Description	Band	Traps (no.)	Trapped area (km^2^)	*p*(capture)
Standard full grid	N/A	1660	209.8	0.71 ± 0.002*
Individual bands	Core	100	2.59	0.67 ± 0.003
	2	392	20.7	0.09 ± 0.003
	3	400	41.4	0.00 ± 0.000
	4	480	62.2	0.00 ± 0.000
	5	288	82.9	0.00 ± 0.000
Uniform density grid	N/A	8100	209.8	0.74 ± 0.002

*mean ± 95 percent confidence limits.

In the leek moth grid, the core had *p*(capture) = 0.85, which was nearly 99 percent of *p*(capture) for the whole grid (above). When solitary, Band 2 had *p*(capture) = 0.03, and Band 3 had *p*(capture) = 0.0.

#### Density effects

**Figure 3:**
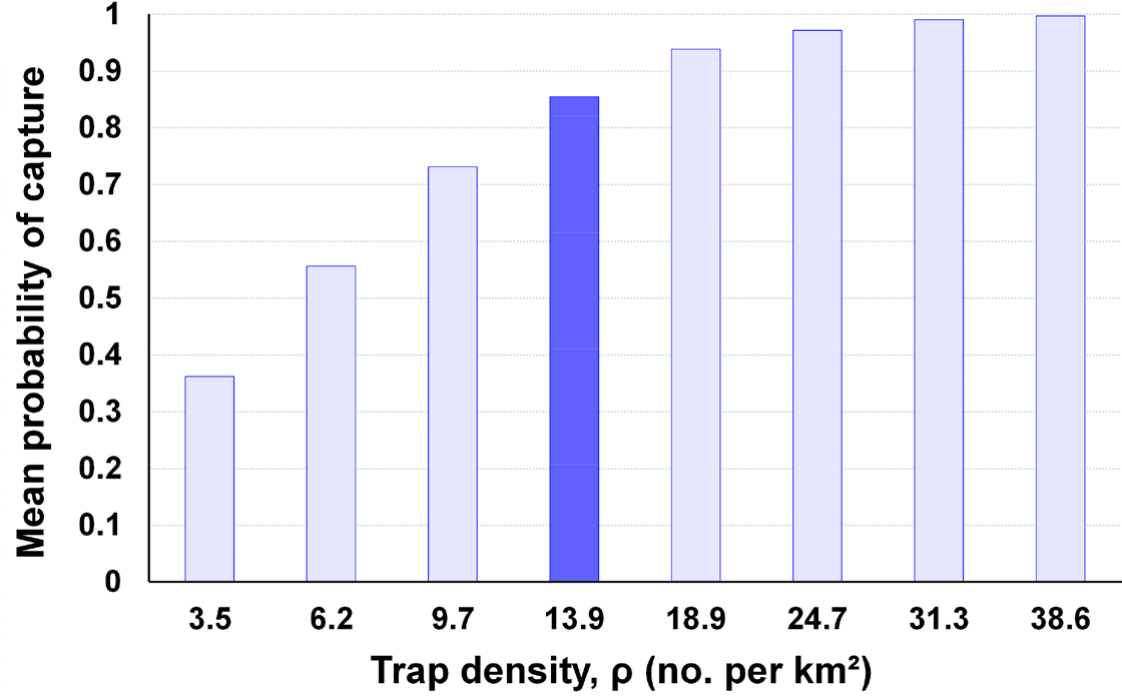
Mean probability of capture for the leek moth grid across different densities. The default density is shown by the dark blue bar.

**Figure 4:**
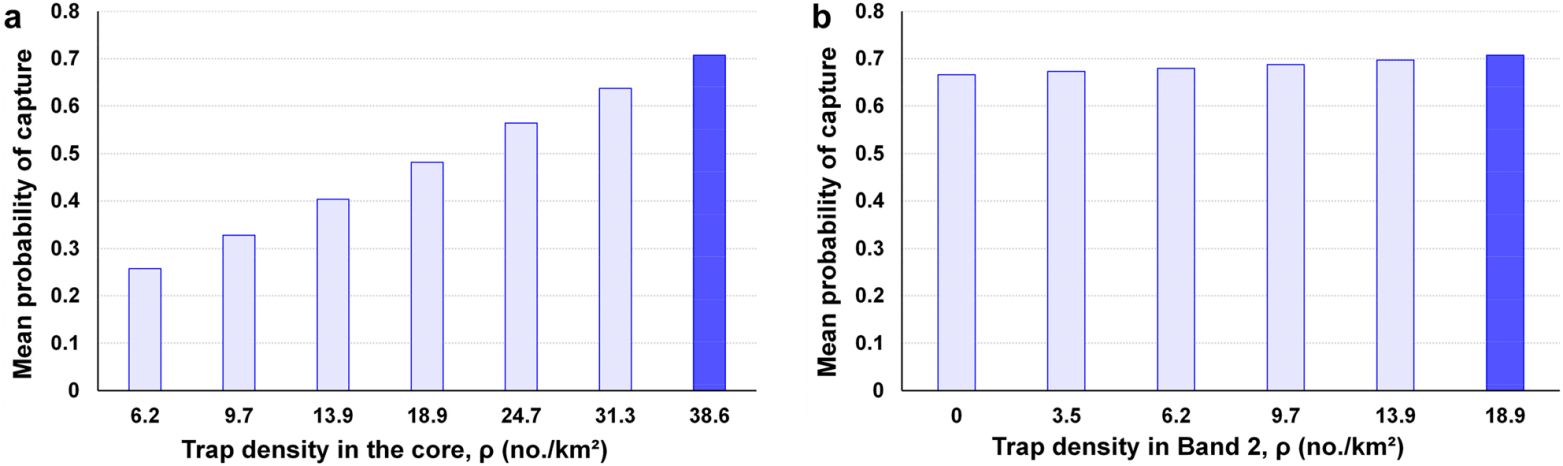
Probability of capture for the entire 14.5-km grid for Medfly across different trap densities in either (**a**) the core, or (**b**) Band 2. Densities in other bands were held constant at default values. Dark blue bars show default values for that band.

All else being equal, *p*(capture) increased with greater trap density, but the marginal increases were smaller at higher densities (Figs. 3, 4). For leek moth, *p*(capture) was only 0.36 at 3.5 traps per km^2^, and it reached around 0.97 at 24.7 traps per km^2^ (Fig. 3).

For Medfly, reducing the core density decreased *p*(capture) markedly for each increment, to a low of 0.26 at 6.2 traps per km^2^ (Fig. 4a). Conversely, reducing the density of Band 2 caused only very small decreases in overall *p*(capture) (Fig. 4b). The total decline in overall *p*(capture) when Band 2 densities dropped from 18.9 to zero traps per km^2^ was only 0.04 (= 0.71 – 0.67). Reducing densities in Bands 3 to 5 had negligible effects on overall *p*(capture) (not shown, but see Table 1). Finally, using the core density throughout the entire grid only increased *p*(capture) by 0.03 compared to the standard design (Table 1).

### Population containment

The value of *D* for leek moth was an order of magnitude less than that for Medfly, which broadly justified the much smaller grid size for leek moth. However, grid sizes for both species were still too large based on their respective *D* values.

#### Size appropriateness of the leek moth grid

The perimeter test indicated that over the 30-d survey duration the likelihood of leek moth egressing from the 8-km grid was zero. This confirmed the results from the band contribution tests above. Consequently, results suggest that a 4.8-km grid was adequate.

#### Size appropriateness of the Medfly grid

The perimeter test for Medfly indicated that the likelihood of egress from the standard grid over 30 days was zero. The band contribution results showed that, for insects with *D* ≤ 50,000, *p*(capture) values in Bands 3, 4, and 5 were zero (Table 1). Since Bands 3 through 5 did not contribute any *p*(capture) at all (Table 1), in a 30-d survey they could be eliminated for Medfly and insects with similar dispersal abilities, resulting in a 4.8-km grid.

### Optimal grid designs

#### Leek moth

The standard grid with 900 traps had a total cost of $25,200. Given *p*(capture) of 0.85, this design achieved 3.4 percentage points per $1,000 spent (= 85/25.2).

Based on the egress likelihood and grid size tests above, we tested a 4.8-km diameter grid for leek moth, because that was adequate for containment. The 4.8-km grid and the circular 8.0-km diameter grid had equivalent *p*(capture) values (Table 2).

**Table 2:** Comparison of circular grids with diameters of either 4.8 km or 8.0 km for leek moth with highly attractive traps (1/λ = 20 m), showing numbers of traps, costs and return on investment (ROI), and the likelihood of capture (*p*(capture)).

Trap density (no./km^2^)	4.8 km diameter grid				8.0 km diameter grid				
	Traps (no.)	Cost ($)	*p*(capture)	ROI*	Traps (no.)		Cost ($)	*p*(capture)	ROI
6.2	112	3,136	0.56	17.9	316		8,848	0.56	6.3
9.7	177	4,956	0.73	14.7	489		13,692	0.73	5.3
13.9	256	7,168	0.86	12.0	716		20,048	0.85	4.3
18.9	349	9,772	0.93	9.6	967		27,076	0.94	3.5
24.7	448	12,544	0.97	7.7	1268		35,504	0.97	2.7
31.3	577	16,156	0.99	6.1	1589		44,492	0.99	2.2
38.6	714	19,992	1.00	5.0	1972		55,216	1.00	1.8

*ROI: return on investment, capture percentage for each $1,000 spent.

**Figure 5:**
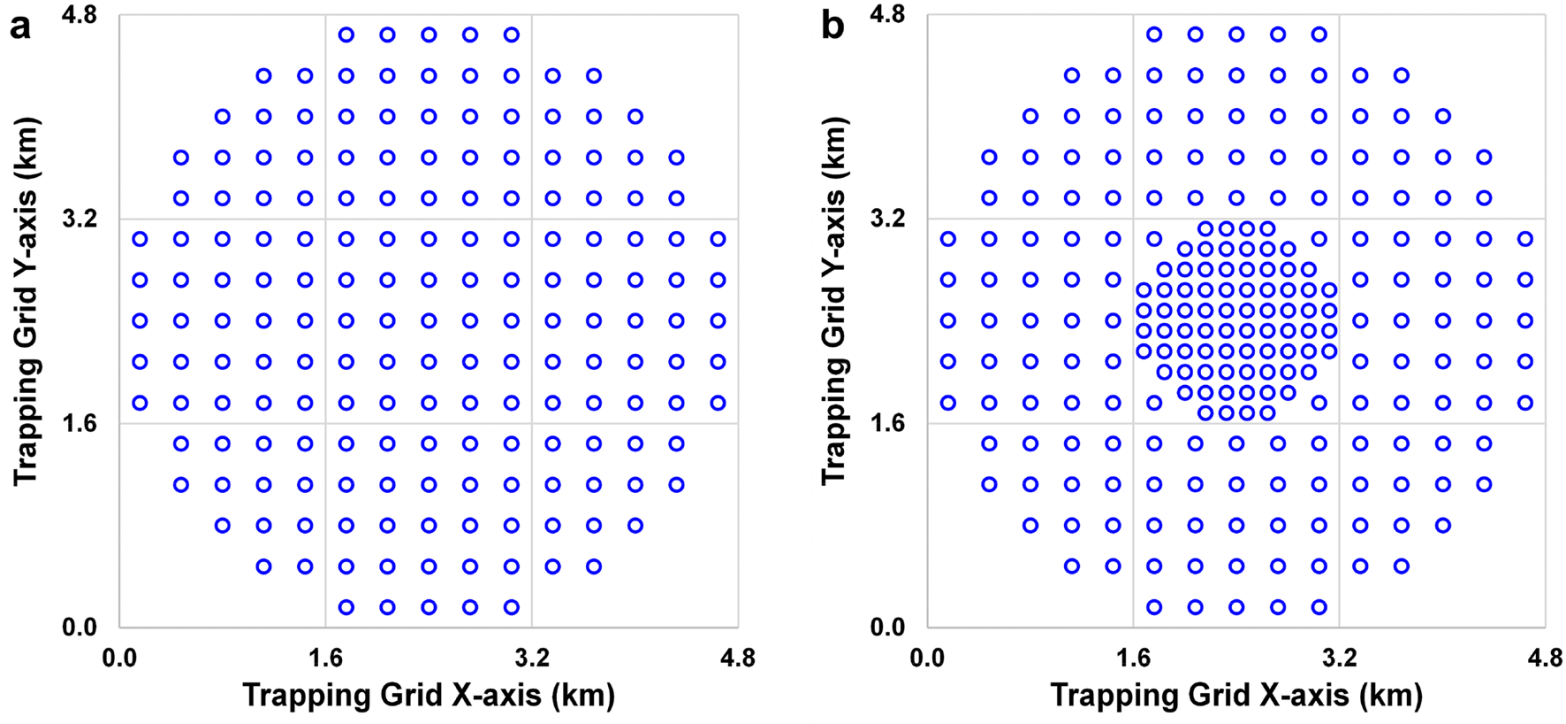
Improved designs for delimiting surveys for (**a**) leek moth, using a 4.8-km diameter circle with 9.7 traps per km^2^, and (**b**) Medfly, with a 4.8-km diameter circle with 38.6 traps per km^2^ in the core and 9.7 traps per km^2^ elsewhere.

As expected, trap density strongly affected *p*(capture). At the default value for 1/λ, *p*(capture) increased from 0.56 at 6.2 traps per km^2^ to 0.85 with 13.9 traps per km^2^ (default). It reached 1.0 with 38.6 traps per km^2^ for both grid sizes. However, because ROI declined as density increased (Table 2) an intermediate density seemed preferable. We judged that the best-performing density was 9.7 traps per km^2^ (Fig. 5a), which achieved *p*(capture) = 0.73 with only 177 traps, giving it an ROI of 14.7 percentage points per $1,000 spent (= 73/4.96). The total survey cost of $4,956 was 80 percent smaller than that for the standard design (Table 2). At that density, increasing the grid size to 8 km would not give any marginal benefit since the *p*(capture) was the same.

Still using the default value for 1/λ, the best performing leek moth grid based solely on ROI was a 4.8-km diameter circular grid with 6.2 traps per km^2^. That design used only 112 traps, cost $3,136, still gave *p*(capture) = 0.56, and had ROI equal to 17.9 percentage points per $1,000 spent (Table 2). Although *p*(capture) for this design was lower, the design was still “more likely than not” to detect the population. Thus, this design might be particularly useful when survey budgets are very small.

If leek moth traps were less attractive than expected, so that 1/λ was equal to only 10 m, then the optimal design would use greater densities than above, but would still cost much less than the standard design. As above, grid size had no effect on *p*(capture) (Table 3). In the 4.8-km grid, the density required to achieve *p*(capture) > 0.5 was 24.7 traps per km^2^. That design used 448 traps and had ROI of 4.6 percentage point per $1000 spent (= 57/12.5), but the cost of $12,544 was half that of the standard design. Much reduced *p*(capture) and ROI compared to the above results (Table 2) also demonstrated how strongly trap attractiveness affects survey effectiveness.

**Table 3:** Comparison of circular grids with diameters of either 4.8 km or 8.0 km for leek moth with less attractive traps (1/λ = 10 m), showing numbers of traps in the design, costs, the likelihood of capture (*p*(capture)), and return on investment (ROI).

Trap density (no./km^2^)	4.8 km diameter grid				8.0 km diameter grid				
	Traps (no.)	Cost ($)	*p*(capture)	ROI*	Traps (no.)		Cost ($)	*p*(capture)	ROI
6.2	112	3,136	0.20	6.3	316		8,848	0.19	2.2
9.7	177	4,956	0.28	5.7	489		13,692	0.28	2.1
13.9	256	7,168	0.38	5.3	716		20,048	0.38	1.9
18.9	349	9,772	0.48	5.0	967		27,076	0.49	1.8
24.7	448	12,544	0.57	4.6	1268		35,504	0.57	1.6
31.3	577	16,156	0.67	4.1	1589		44,492	0.67	1.5
38.6	714	19,992	0.74	3.7	1972		55,216	0.74	1.3

*ROI: return on investment, capture percentage for each $1,000 spent.

#### Medfly

The standard Medfly grid with 1,700 traps (variable densities) would cost $44,200 for a 30-d survey. This gave ROI of only 1.6 percentage points per $1,000 spent (= 71/44.2) (Table 4).

**Table 4:** Comparison of alternative delimiting survey design options for Medfly , for circular grids with different diameters and trap densities, showing numbers of traps, costs and return on investment (ROI), and the likelihood of capture (*p*(capture)).

Design	Grid size	Outer bands density	Total traps	*p*(capture)	Cost		ROI^‡^
	(km)	(no./km^2^)*	(no.)		Total ($)	Reduction^†^	
Standard	14.5	Variable (default)	1,700	0.71	44,200	–	1.6
Alternative 1	4.8	Variable (default)	392	0.70	10,192	0.77	6.9
Alternative 2	4.8	13.9	304	0.68	7,904	0.82	8.6
Alternative 3	4.8	9.7	232	0.66	6,032	0.86	10.9
Alternative 4	6.4	6.2	272	0.65	7,072	0.84	9.2

*All alternatives had the same density of 38.9 traps per km^2^ in the core.

^**†**^ Reduction relative to the standard survey cost.

^**‡**^ ROI: return on investment, capture percentage points for each $1,000 spent.

Every alternative tested had similar *p*(capture) as the standard design, but much greater ROI, because of the smaller grid sizes (Table 4).

The best design overall was Alternative 3 (Fig. 5b), which had *p*(capture) = 0.66, used 232 traps, and only cost $6,032, which saved $38,168 (86 percent; Table 4). It gave ROI of 10.9 percentage points per $1,000 spent (= 66/6.03), which was nearly seven times greater than the ROI for the standard design.

Alternative 1, which used default densities in a smaller grid, gave *p*(capture) = 0.70 (Table 4). It used 392 traps and would cost $10,192, a savings of $34,008 (77 percent). Its ROI was 6.9 percentage points per $1,000 spent (= 70/10.19), which was more than 4 times greater than that for the standard grid.

Alternative 2 used a greater trap density than Alternative 3. It required 304 traps and gave *p*(capture) = 0.68 (Table 4). The design would cost $7,904, saving $36,296 (82 percent), with ROI of 8.6 percentage points every $1,000 spent (= 68/7.9).

Given the high-risk pest status of Medfly, some managers might choose a larger 6.4-km grid. We tested this with a much reduced trap density as Alternative 4. That design achieved *p*(capture) = 0.65, used 272 traps, and would cost $7,072, a savings of $37,128 (84 percent reduction) (Table 4). The ROI for this design was 9.2 percentage points per $1,000 spent (= 65/7.07), which was six times greater than that for the standard Medfly grid.

## Discussion

### Key delimitation trapping survey performance factors

#### Trap attractiveness

The performance of the current Medfly design was unexpectedly inferior to that of the leek moth even with a more vagile target insect, 2.8 times greater trap density in the core, and a grid size over three times larger. Despite all those factors, *p*(capture) for the leek moth grid with 1/λ = 20 m was 15 percentage points greater than that for Medfly at 30 days duration. Thus, trap attractiveness was the key determinant for delimiting survey performance, as it was for detection^13^.

One straightforward way to improve *p*(capture) and the accuracy of boundary setting, while also cutting costs, would be to develop more attractive traps. Poorly attractive traps include food-based attractants^48^ and traps based solely on visual stimuli^36^. But developing better traps is difficult. Pheromone-based attractants generally perform best^49^, but these are unavailable for many insects. For instance, scientists have searched for decades for effective pheromones for *Anastrepha suspensa* (Loew) and *A. ludens* (Loew) without success^50^. Common issues include the complexity of components, costs of synthesis, and chemical stability.

#### Trap densities

All else being equal, increasing the trap density will generally improve *p*(capture) for any survey grid, and intuitively this can help compensate for using less attractive traps. However, the impact of increasing density is limited when attractiveness is low^13,47^, and large surveys or grids with many traps can become prohibitively expensive^51^. The Medfly grid designers likely understood that the available trap and lure was not highly attractive, and used higher densities in inner bands to try to reach some desired (non-quantitative) survey performance level. By contrast, the designers of the leek moth grid used a (constant) density three times smaller, likely because the trap and lure were known to be relatively strong. Here, for both species, marginal ROI decreased as densities increased (Tables 2, 3). Hence, increasing densities has limited benefit, but may be useful when better lures are unavailable^13^.

In that context, the use of variable densities in the Medfly grid is understandable. At its standard size, the survey grid would require 8,100 traps if the core trap density were constant (Table 1). The designers likely intuited that lower densities could be used in outer bands because captures there were less likely. However, doing so reduces the likelihood of detection in outer bands and could increase the possibility of undetected egress, especially with longer survey durations. As far as we know, natural egress has not been raised as a concern following the numerous Medfly quarantines that have used this survey grid over the years, in Southern California in particular^52^.

Generally, however, we think the variable Medfly grid densities run counter to delimitation goals. Greater core and Band 2 densities have proportionally more impact on *p*(capture), but only a few detections in the core are necessary to confirm the presence of the population (Goal 1), and inner area detections probably contribute little to boundary setting (see below). Therefore, lower or intermediate densities (at most) may be optimal for the core when considering ROI. For the outer bands, increasing densities might improve boundary setting (Goal 2) and help mitigate potential egress, but the sizes of those bands already limit cost efficiency (Table 2), making greater densities less advisable. Our simulation results can help elucidate how to balance these interests to achieve delimitation goals while minimizing costs^47^.

### Grid size considerations

The simulation results indicated that the standard survey sizes for these two pests were excessive. We have verified that empirically for Medfly using trapping detections data^53^. A 14.5-km grid has been widely used for many other insects in the CDFA (2013) guidelines^10^, such as Mexfly and OFF, and the same analysis indicated that those are also oversized for use in short-term delimitation surveys^53^. From the same analysis, the predicted survey radius for leek moth, with *D* = 500 m^2^ per day, would be 2,382 m, or a diameter of nearly 4.8 km, which matches the results here. Similarly, Dominiak and Fanson^45^ analyzed trapping data for Qfly and found that the recommended quarantine area distance of 15 km could be reduced to 3 to 4 km.

Grids with radii larger than 4.8-km only seem necessary for highly vagile insects, those with *D* ≥ 50,000 m^2^ per day^47^. This should not be surprising. Small insect populations are unlikely to move very far^31,54^, especially if hosts are available^20,39,55^. The (proposed) short duration of a delimitation survey would also limit dispersal potential (see below). Many delimiting survey plans may be oversized, because they were developed before much dispersal research had been done^37^, thus uncertainty was high. Our dispersal distance analysis included species with a wide range of dispersal abilities, so it can be used generally to choose smaller survey grid radii^53^.

Reducing grid sizes down to about 4.8-km diameters may have little impact on *p*(capture), since detections in bands outside that distance contributed little to overall performance. The cores of both the leek moth and Medfly grids accounted for 86 percent or more of overall *p*(capture). While core area detections will confirm the presence of the population, they are less useful for defining spatial extent. The furthest detections from the presumed source are usually used to delimit the incursion^46,56^ (although in our experience formal boundary setting exercises seem rare). Delimiting surveys may often yield few captures anyway, because adventive populations can be very small and subject to high mortality^31^. Because size reductions eliminate traps in proportionally larger outer areas, the impact on survey costs is substantial. Removing just the outermost bands of each grid would directly reduce costs by $11,200 for leek moth (400 traps) and by $7,488 for Medfly (288 traps; Table 1).

Another reason for the large size of the standard Medfly grid may be that it was designed for monitoring and management in addition to delimitation^57^. Medfly quarantines end after at least three generations without a detection, so the surveys may last for months. The grid size was reportedly originally determined by multiplying the estimated dispersal distance by three (PPQ, personal communication), to account for uncertainty. This implies that the estimated distance was about 2,400 m per 30 days. Thus, the design may not have been built for the 30-d duration used here, but our recommended design is valid if a shorter delimitation activity without further monitoring is appropriate.

Although it seemed too large for leek moth, an 8-km grid for delimitation could be appropriate for some other moths. For example, the delimiting survey plans for *Spodoptera littoralis* (Boisduval) and *S. exempta* Walker use this size^9^. *S. littoralis* is described as dispersing “many miles”, and *S. exempta* can travel hundreds of miles^9^, which clearly exceeds the described dispersal ability of leek moth. On the other hand, the survey plan for summer fruit tortrix moth (*Adoxophyes orana* Fischer von Röeslerstamm) also specifies an 8-km grid for delimitation but contains little information on dispersal, suggesting only that most movement is local^8^. Like leek moth, a 4.8-km grid for that species seems likely to be more appropriate.

Limiting egress potential is probably the main consideration when setting survey size, but uncertainty about the source population location may also be a factor. Survey grids placed over the earliest insect detection may sometimes be off center from the location of the source population^54^. However, so far as we know for our agency, most adventive populations have been localized, based on post-discovery detections (PPQ, personal communication). Likewise, we have found^53^ and other researchers have found that dispersal distances for different species in outbreaks and mark-recapture studies are often less than 1 km^58–60^. That may often be the case for detection networks of traps (e.g., for high risk fruit flies), which increase the likelihood of capture before the population has had much time to grow and disperse. Here, we focused explicitly on localized populations, but allowed for uncertainty in the simulations by varying outbreak locations over one mile in the central part of the grid. If the outbreak population is very large and has extensively spread out (e.g., spotted lanternfly, *Lycorma delicatula* (White) in 2014^61^), delimitation will not be localized, but “area-wide”^2^. The results here do not apply to area-wide outbreaks, and we are currently studying how to effectively delimit them.

### Optimizing delimitation surveys

Many trapping survey designs in use were based not on “hard” science but on local experience^62^. Scientists have recognized the need for more cost-effective surveillance strategies^63,64^. Quantitatively assessing *p*(capture) in different designs for the same target pest allows us to determine grid sizes and densities that lower costs while maintaining performance. Results here demonstrated that the sizes and densities of these two survey grids could be optimized to save up to $20,244 per survey for the leek moth and $38,168 per survey for the Medfly. In practical terms, that means more than five leek moth surveys could be run for the cost of one standard design survey. Additionally, over seven Medfly delimitation surveys could be funded by the budget of one standard plan. The magnitudes of reduction seen here may be typical, since about 90 percent of the costs in trapping surveys are for transportation and maintenance related to traps^65^.

Quantifying survey performance was not possible until very recently, so it has been little discussed in the literature^5,66^, and no standard thresholds exist. We think 0.5 may be a reasonable minimum threshold for the choice of *p*(capture), to try to ensure that population detection is “more likely than not”. Designs that aim to maximize *p*(capture) could be realistic with high attractiveness traps, but those designs seem very likely to have lower ROIs (e.g., Table 2). Even for the most serious insect pests, we think targeting near-perfect population detection during delimitation is likely not justified. Designs achieving *p*(capture) from 0.6 to 0.75 could be highly effective in terms of both costs and performance.

Another potential area of improvement is grid shape. Circular grids perform as well as square grids but use fewer traps and less service area to achieve equivalent *p*(capture)^47^. Moreover, detections in the corners of a square grid are evidence that insects could have traveled beyond the square along the axes, resulting in uncertain boundary setting. Most published survey grids are square^10,46^, but many field managers tend to use approximately circular trapping grids in the field (PPQ, personal communication). The conversion to a circular grid with a radius of half the square side length reduces the area and number of traps by around 21 percent^47^. Our findings were consistent with that value.

This new quantification ability also indicates that some delimiting survey designs in the U.S.A. may not be performing as well as expected^47^. For instance, the delimiting survey design for Mexfly uses approximately 31 traps per km^2^ in the core of a 14.5 km square grid^11^, but the traps are only weakly attractive (1/λ ≈ 5 m). In this scenario, *p*(capture) was only around 0.23 with a 30-d survey duration^47^. A much greater density (> 80 traps per km^2^) could be used in the core to achieve *p*(capture) ≥ 0.5, but this may not be feasible depending on the survey budget.

### Technical and modeling considerations

Examining diffusion-based movement for these two insects in TrapGrid can give insight into why simulations indicated that smaller grids may be adequate^47^. The value of σ for Medfly after 30 days is only about 1,550 m. In a normal distribution, σ = 1,550 m gives a 95th percentile distance of 2,550 m, which is similar to the estimated distance above of 2,400 m. Over 90 days, σ = 2,700 m for Medfly, which gives a 95th percentile distance of 4,441 m, still much shorter than the grid radius of 7,250 m. A 95th percentile of 7,250 m requires σ ≈ 4,408 m, which equals *t* = 253 days. In addition, the maximum total distance (up to 39 days after detection) we observed in trapping detections data for Medfly in Florida was about 4,800 m^53^.

The same calculations for leek moth give σ ≈ 490 m for 30 days, with a 95th percentile distance of only 806 m. That is half the length of the recommended shortened radius above of 2.4 km, and nearly five times shorter than the radius of the standard 8-km grid. A 95th percentile of 4,000 m requires σ = 2,432 m, which implies *t* = 740 days, which is about two years. Therefore, the leek moth grid is arguably even more oversized than the Medfly grid.

The default capture probability calculation in the current version (Ver. 2019-12-11) of TrapGrid is not sensitive to population size^32^ and does not consider the effects of ambient factors (e.g., wind speed and direction, rainfall, temperature). Many other factors can also impact trapping survey outcomes, such as topography of the environment, availability of host plants, seasonality of pest, and population dynamics. These factors are not considered in the current version of TrapGrid.

## Conclusions

We used TrapGrid simulations to isolate different survey design factors in the two grids and demonstrated the utility of the model to investigate alternatives that would be costly and time-consuming to test in the field. It may not be possible to use TrapGrid to optimize delimiting survey designs for *all* combinations of dispersal abilities and trap attractiveness levels because of the very large number of simulations that would require, and because the empirical parameters for many insects and trap combinations are unknown. However, our results demonstrated that such optimization is possible for select insects, and TrapGrid has performed well with two studied species^13^. General information on selecting trap densities based on attractiveness is available^47^. The accuracy of the model with regard to recommending smaller grid sizes has now also been empirically verified^53^. We recommend using targeted field experiments to validate simulation results that might be of interest to programs.

## Data Availability

The model results generated and analyzed during the current study are available from the corresponding author on reasonable request.
